# Biochemical Pathways in Cancer 

**DOI:** 10.1155/2012/268504

**Published:** 2012-11-20

**Authors:** Eun-Kyoung Yim Breuer, Mandi M. Murph, Rolf J. Craven

**Affiliations:** ^1^Department of Radiation Oncology, Loyola University Chicago, Maywood, IL 60153, USA; ^2^Department of Pharmaceutical and Biomedical Sciences, University of Georgia, Athens, GA 30602, USA; ^3^Department of Molecular and Biomedical Pharmacology, University of Kentucky, Lexington, KY 40506, USA

The revolutionary advances in cellular and molecular biochemistry in the last quarter century have provided an unprecedented opportunity for systematic approaches to understand the cancer process. With these new advances and the knowledge acquired as a direct result, an enormous impact has positively affected the clinical areas of cancer prevention, disease management, and treatment of malignancy. Despite these scientific achievements, we are still faced with the challenge of fully understanding the complex nature of cancer, including issues of disease recurrence and drug resistance. Therefore, dissecting biochemical pathways that underlie the development of cancer should be given high priority to improve knowledge of human health. 

 In this special issue, we will explore the various aspects of cancer-related biochemical pathways. The papers in this issue discuss multiple signaling pathways involved in the regulation of proliferation, senescence, and death of cancer cells. M. K. Altman et al. demonstrated that the regulator of G-protein signaling 5 (RGS5) is able to reduce the proliferation of ovarian cancer cells and extend the survival of mice. The tumor suppressor p53 plays an essential role in cell proliferation, cell cycle progression, cell death, and senescence [1, 2]. Reisman et al. reviewed the evidence that DNA-binding factors RBP-J*κ* and C/EBP*β*-2 transcriptionally activate p53 to ensure a rapid cellular response to DNA damage. p53 is also involved in complex networks of mitotic kinase signaling in response to mitotic spindle damage to ensure the proper cell cycle progression. G. H. Ha and E. K. Breuer discussed a mutual regulation network between mitotic kinase and p53 signaling. The tumor suppressor p16^INK4A^ has shown to be implicated in replicative senescence. Here, the role of p16^INK4A^ in replicative senescence and DNA damage-induced premature senescence in the absence of p53 was reviewed by R. Mirzayans et al. The authors also discussed a possible existence of a negative regulatory relationship between p53 and p16^INK4A^. R. Jäger and H. O. Fearnhead reviewed cancer cell proliferation and death in a different point of view. These authors suggested that cancer cell proliferation might be a consequence of inappropriate or corrupted tissue-repair programs, initiated by a signal from dying cells. Mounting evidence suggested that dissecting the biochemical process of genetic alterations will help us understand mechanisms underlying the genesis and spread of cancer and develop novel strategies for targeted therapy and tailored cancer management [3, 4]. J. L. Johnson et al. discussed the genetic and biochemical alternations in heterogeneous cell signaling pathways in non-small-cell lung cancer and its implications in targeted cancer therapy. The papers also provide new insights into the pathogenesis of cancer and therapeutic intervention strategies. As a new therapeutic target, S. Mohanan et al. suggested that peptidylarginine deiminases (PADs) play an important role in cancer pathogenesis and its inhibition may have a profound impact on the regulation of the inflammatory tumor microenvironment and tumor growth. To improve the efficacy of chemotherapy in hormone-refractory prostate cancer, E. Tsakalozou et al. investigated the combination effects of docetaxel and doxorubicin and proposed the synergistic ratios of drug combinations.

 We believe that a better understanding of cellular biochemical pathways and activities will ultimately have important implications in the management of cancer patients. Previous work in this area has demonstrated the power of translating molecular and biochemical findings to therapeutics and the clinic. It is our hope that the papers in this issue will be of great help in understanding the complex nature of cancer and in designing and conducting preventive and therapeutic interventions in the near future.

*Eun-Kyoung Yim Breuer* *Mandi M. Murph* *Rolf J. Craven*
